# Hybrid Graphene Oxide/Ion-Imprinted Polymer via Single-Step Grafting–Imprinting for High-Performance and Selective Cu(II) Adsorption

**DOI:** 10.3390/polym18111362

**Published:** 2026-05-30

**Authors:** Pablo Carmona, María Gabriela Lobos, Gonzalo Riveros, Rodrigo Segura, Monserrat Olivares, Pamela Lazo

**Affiliations:** 1 Instituto de Química, Universidad de Valparaíso, Avenida Gran Bretaña 1111, Playa Ancha, Valparaíso 2360102, Chile; gabriela.lobos@uv.cl (M.G.L.); gonzalo.riveros@uv.cl (G.R.); rodrigo.segura@uv.cl (R.S.); mo.thays.od@gmail.com (M.O.); pamela.lazo@uv.cl (P.L.); 2 Laboratorio de Química Analítica y Ambiental, Facultad de Ciencias, Universidad de Valparaíso, Avenida Gran Bretaña 1111, Playa Ancha, Valparaíso 2360102, Chile; 3 Laboratorio de Electroquímica, Facultad de Ciencias, Universidad de Valparaíso, Avenida Gran Bretaña 1111, Playa Ancha, Valparaíso 2360102, Chile; 4 Laboratorio de Nanomateriales, Facultad de Ciencias, Universidad de Valparaíso, Avenida Gran Bretaña 1111, Playa Ancha, Valparaíso 2360102, Chile; 5 Centro de Observación Marina para Estudios de Riesgos del Ambiente Costero (COSTA-R), Facultad de Ciencias, Universidad de Valparaíso, Avenida Gran Bretaña 1111, Playa Ancha, Valparaíso 2360102, Chile

**Keywords:** ion-imprinted polymers, graphene oxide, hybrid materials, Cu(II) adsorption, surface imprinting, grafting–imprinting polymerisation

## Abstract

The increasing release of Cu(II) into aquatic environments has intensified the demand for efficient and selective removal strategies. Although adsorption is widely applied for Cu(II) removal, its performance is often constrained by limited accessibility and low selectivity of active sites. In this study, a hybrid ion-imprinted polymer was synthesised via a single-step grafting–imprinting–polymerisation (SGPI) strategy, enabling the formation of a surface-oriented imprinted polymer layer on a functionalised graphene oxide support (GO/MPS). 4-vinylpyridine (4VP) was employed as the functional monomer to promote specific Cu–N coordination and facilitate binding-site formation. The resulting GO/MPS@IIPs-Cu(II) achieved an adsorption capacity (Qmax) of 256 mg g^−1^, together with faster adsorption kinetics relative to bulk IIPs-Cu(II). The material also demonstrated improved selectivity for Cu over competing ions (Co, Fe, and Ba), as well as satisfactory reusability, maintaining extraction efficiencies above 98% after eight adsorption–desorption cycles. These findings demonstrate that the SGPI strategy enables a more organised distribution of imprinted binding sites, thereby improving their accessibility and promoting a synergistic combination of high adsorption capacity, rapid kinetics, selectivity, and reusability. This approach establishes a robust platform for the development of advanced hybrid ion-imprinted polymers for the selective removal of metal ions.

## 1. Introduction

The expansion of energy-transition technologies has significantly increased the global demand for copper (Cu), a metal essential for electrical, electronic, and renewable-energy infrastructures [1,2,3]. At the same time, intensified extraction, refining, and industrial use have led to elevated Cu(II) concentrations in aqueous systems [4,5,6]. In such environments, Cu(II) speciation is strongly influenced by pH, inorganic ligands, and natural organic matter, promoting the formation of complexes and aggregates that complicate its removal [7,8].

Various technologies have been investigated for Cu(II) removal, including cementation, membrane filtration, electrochemical treatments, and photocatalysis [7,8,9,10]. However, these approaches often suffer from high energy consumption, secondary waste generation, or reduced efficiency in complex media [9,10]. Adsorption has therefore emerged as a versatile alternative. Nevertheless, its performance is strongly governed by the accessibility, distribution, and selectivity of active sites, as well as the structural stability of the adsorbent during repeated use.

Ion-imprinted polymers (IIPs) have attracted considerable attention due to their ability to generate recognition sites with specific affinity for target ions [11]. These materials are typically synthesised through the formation of a complex between a template ion and a functional monomer, followed by polymer crosslinking. After template removal, cavities with complementary geometry and coordination environments are formed, enabling selective recognition [12]. The nature of the functional monomer plays a central role, as its donor groups define the coordination environment of the metal ion during imprinting [13]. Although various strategies have been reported for Cu(II) imprinting, including the use of copolymers and auxiliary ligands [14,15], improvements in selectivity do not necessarily translate into increased adsorption capacity, particularly when binding sites are poorly accessible or weakly defined [16].

Cavity accessibility is a key factor limiting IIP performance. Conventional polymerisation often produces highly crosslinked and compact structures in which binding sites may be partially occluded, resulting in reduced accessibility and slower adsorption kinetics [17]. Surface imprinting strategies have been proposed to overcome this limitation by localising recognition sites near the material surface. However, these approaches frequently involve multistep synthesis and may lead to heterogeneous coatings, affecting reproducibility and the effective number of accessible sites [18,19].

To address these limitations, synchronous grafting, imprinting, and polymerisation (SGPI) has been proposed as an alternative strategy that enables the simultaneous anchoring of the metal–monomer complex onto a support and the formation of the polymer network in a single step [20,21,22]. This approach promotes surface-oriented growth and improves the accessibility and exposure of imprinted sites compared with bulk IIPs. Nevertheless, its effectiveness depends strongly on the nature of the support, which influences grafting efficiency and cavity structure [23,24].

Graphene oxide (GO) is a promising support due to its high surface area, two-dimensional structure, and abundance of oxygen-containing functional groups, which facilitate further functionalisation [25,26]. GO-based IIP systems have demonstrated improved adsorption performance, particularly when combined with surface imprinting strategies [27,28]. In this context, functionalisation with 3-(trimethoxysilyl)propyl methacrylate (MPS) introduces polymerisable vinyl groups onto the GO surface, enabling controlled grafting and promoting the exposure of imprinted binding sites [29,30].

Despite these advances, SGPI-based GO/MPS-supported Cu(II) IIPs using N-donor monomers such as 4-vinylpyridine (4VP) remain scarcely explored. In this work, a hybrid material (GO/MPS@IIPs-Cu(II)) was synthesised via an SGPI strategy to improve the accessibility and distribution of imprinted binding sites. The material was characterised by FTIR, Raman, FESEM, and EDS, and its adsorption behaviour was evaluated through pH-dependent, kinetic, isotherm, selectivity, and reusability studies. This study provides insights into the role of synchronous polymerisation and support functionalisation in controlling polymer architecture and improving the performance of ion-imprinted polymers.

## 2. Materials and Methods

### 2.1. Materials

Ultrapure water (18.2 MΩ·cm), used for all solutions, was obtained from a Milli-Q Synergy^®^ system (Merck Millipore, Burlington, MA, USA). All experiments were performed at room temperature (20 ± 1 °C). The following reagents were purchased from Sigma-Aldrich (St. Louis, MO, USA): 3-(trimethoxysilyl)propyl methacrylate (MPS, 98%), ethylene glycol dimethacrylate (EGDMA, 98%), azobisisobutyronitrile (AIBN, 0.2 M in toluene), 4-vinylpyridine (4VP, 95%), and graphite powder (325 mesh, 99%). Glacial acetic acid, nitric acid (65%), sulfuric acid (98%), hydrogen peroxide (30%), copper(II) nitrate trihydrate, methanol, sodium nitrate, potassium permanganate, monopotassium phosphate, and dipotassium phosphate were obtained from Merck (Darmstadt, Germany). All reagents were of analytical grade and were used as received.

### 2.2. Instrumentation

Metal ion concentrations were determined by inductively coupled plasma optical emission spectrometry (ICP-OES, iCAP PRO Thermo Fisher Scientific, Waltham, MA, USA). Raman spectra were recorded using a Renishaw inVia Raman microscope (Renishaw, Gloucestershire, UK) equipped with a 532 nm laser. Morphological analysis and elemental composition were evaluated using a Thermo Scientific Quattro S field-emission scanning electron microscope (FESEM) coupled with energy-dispersive X-ray spectroscopy (EDS) (Thermo Fisher Scientific, Waltham, MA, USA). FTIR spectra were recorded on a Nicolet iS10 spectrometer (Thermo Fisher Scientific, Waltham, MA, USA). GO and GO/MPS samples were analysed in transmission mode using KBr pellets, while polymer-based materials were measured in ATR mode. Spectra were collected in the range 4000–400 cm^−1^.

### 2.3. Synthesis of Ion-Imprinted Polymers

The IIPs-Cu(II) polymer was synthesised via free-radical polymerisation. A pre-polymerisation complex was prepared by mixing 431 μL of 4VP with 1 mL of a 1 M Cu(NO_3_)_2_ solution in 10 mL of methanol, followed by magnetic stirring for 1 h. Subsequently, 3.77 mL of EGDMA (crosslinker) and 1 mL of AIBN solution (0.2 M in toluene) were added. The mixture was purged with argon for 10 min, sealed, and polymerised at 65 °C for 24 h. The resulting polymer was then filtered, washed thoroughly with deionised water, and dried at 60 °C for 24 h. The dried material was ground and sieved to obtain particles with a uniform size distribution. A non-imprinted polymer (NIP) was prepared following the same procedure without the Cu(II) template ion.

Template removal was carried out using 10% (*v*/*v*) HNO_3_. In each cycle, 1 g of polymer was treated with the acidic solution for 1 h, followed by centrifugation at 5000 rpm. The supernatant was analysed by ICP-OES. Although no Cu(II) was detected after three cycles, the washing procedure was extended to six cycles to ensure complete template removal. No residual Cu(II) was detected after the sixth cycle.

### 2.4. Synthesis of Graphene Oxide (GO)

GO was synthesised from graphite using a modified Hummers’ method. Graphite powder (1 g) and NaNO_3_ (0.5 g) were added to concentrated H_2_SO_4_ (50 mL) under ice-bath conditions (0 ± 1 °C) and stirred for 30 min. KMnO_4_ (3 g) was slowly added while maintaining the temperature below 5 °C with continuous stirring. The mixture was then heated to 30 °C, followed by the dropwise addition of hot deionised water (~80 °C, 50 mL) with continuous stirring. The temperature was then increased to 90 °C and maintained for 1 h. The residual KMnO_4_ was removed by adding H_2_O_2_ (30%, 20 mL). The product was washed with deionised water until pH 6–7 and dried at room temperature.

### 2.5. Functionalisation of GO with MPS

GO was functionalised via silanisation using MPS. GO was dispersed in water by ultrasonication for 15 min prior to silanisation. An acidified methanol–water solution (100 mL, 80:20 *v*/*v*, pH 3.5–4.5) was prepared, MPS was added at a 1:1 (*w*/*w*) ratio relative to GO, and the mixture was stirred for 1 h. The MPS-containing solution was added dropwise, and the mixture was stirred at 65 °C for 2 h. The product was dried at 120 °C for 2 h, washed with a methanol–water mixture and deionised water, and dried at 60 °C for 24 h to obtain the GO/MPS material.

### 2.6. Synthesis of GO/MPS@IIPs-Cu(II)

The hybrid adsorbent was synthesised via an SGPI-based surface imprinting approach. A pre-polymerisation complex was prepared by mixing 431 μL of 4VP with 1 mL of a 1 M Cu(NO_3_)_2_ solution in 5 mL of methanol, followed by stirring for 1 h. In parallel, the GO/MPS material was dispersed in 5 mL of methanol by ultrasonication to maintain a constant total solvent volume of 10 mL. The monomer–template complex was then combined with the GO/MPS dispersion, followed by the addition of 3.77 mL of EGDMA (crosslinker) and 1 mL of AIBN solution (0.2 M in toluene). The mixture was purged with argon for 10 min and polymerised at 65 °C for 24 h. The resulting material was filtered, washed with deionised water, dried at 60 °C for 24 h, ground, and sieved. Variants were prepared using 5, 10, 50, and 100 mg of GO/MPS as the support material. NIPs were synthesised following the same procedure without the Cu(II) template ion.

### 2.7. Adsorption Experiments

Batch adsorption experiments were performed using 30 mg of adsorbent and 20 mL of Cu(II) solution. For pH studies, solutions (20 mg L^−1^) were adjusted over the pH range 2–8. Suspensions were maintained under constant stirring for 1 h, followed by centrifugation and filtration (0.45 μm) prior to ICP-OES analysis. The amount adsorbed at equilibrium (Qe) was calculated according to Equation (1):

(1) $$Qe=\frac{\left(C_{0}-C_{e}\right)V}{W}$$

where C_0_ and C_e_ (mg L^−1^) are the initial and equilibrium concentrations, respectively; V (L) is the solution volume; and W (g) is the adsorbent mass.

Kinetic experiments were conducted at pH 7.0 with an initial concentration of 20 mg L^−1^ over contact times ranging from 5 to 120 min. Adsorption isotherms were obtained using initial concentrations ranging from 10 to 600 mg L^−1^. Kinetic data were fitted to the pseudo-first-order (PFO), pseudo-second-order (PSO), and Elovich models, whereas equilibrium data were fitted to the Langmuir and Sips isotherm models and the Freundlich equation using OriginPro (v.2024). Model selection was based on χ^2^ and R^2^ values, as well as the agreement between experimental and fitted data.

Selectivity experiments were carried out in binary systems (Cu(II)/Co(II), Cu(II)/Fe(III), and Cu(II)/Ba(II)) with initial concentrations of 20 mg L^−1^ for each ion. The distribution coefficient (Kd), selectivity coefficient (k), relative selectivity coefficient (k′), and extraction efficiency (%E) were calculated according to Equations (2)–(5).

(2) $$K_{d}=\frac{\left(C_{0}-C_{e}\right)V}{C_{e}\cdot W}$$

(3) $$k=\frac{K_{d}(Cu\left(II\right))}{K_{d}(M)}$$

(4) $$k^{\prime }=\frac{k_{imprinted}}{k_{non-imprinted}}=\frac{k_{IIPs}}{k_{NIP}}$$

(5) $$\%E=\left(\frac{C_{0}-C_{e}}{C_{0}}\right)\times 100$$

where C_0_ and C_e_ (mg L^−1^) are the initial and equilibrium concentrations, respectively; V (L) is the solution volume; W (g) is the adsorbent mass; and M represents the competing metal ion.

Reusability was evaluated over eight adsorption–desorption cycles using 10% HNO_3_ as the eluent. All experiments were performed in triplicate, and the results are reported as mean values.

ChatGPT 5 (OpenAI, accessed 15 April 2026) was used to assist in the conceptualisation of the graphical abstract. All generated content was critically reviewed and edited by the authors.

## 3. Results and Discussion

### 3.1. Mechanisms of Synthesis of IIPs-Cu(II) and Hybrid GO/MPS@IIPs-Cu(II)

The ion-imprinted polymer IIPs-Cu(II) was synthesised using 4-vinylpyridine (4VP) as the functional monomer, selected for its ability to coordinate Cu(II) through the pyridinic nitrogen, in accordance with the Hard and Soft Acids and Bases (HSAB) principle [31]. In contrast to oxygen-donor monomers (e.g., acrylic or methacrylic acid) and amide-containing monomers (e.g., acrylamide), whose donor atoms are harder and whose electron density is partially delocalised due to resonance effects [32,33], 4VP provides a moderately soft nitrogen donor that favours more stable and directional coordination interactions with Cu(II) [34,35].

Cu(II), as a d^9^ metal centre, typically adopts distorted octahedral coordination geometries with nitrogen-donor ligands, where the Jahn–Teller effect leads to elongated axial bonds and more defined equatorial interactions [36,37,38]. In this context, the pre-polymerisation complex formed with 4VP is expected to promote well-defined coordination environments that can be partially retained within the imprinted cavities after crosslinking, contributing to the formation of selective recognition sites.

During the pre-polymerisation step, 4VP formed a complex with the template ion, which was subsequently crosslinked with EGDMA using a 1:4:20 molar ratio (T:M:C, template:monomer:crosslinker). The resulting bulk IIPs-Cu(II) material was used as a reference to evaluate the effect of the support on site accessibility and adsorption capacity.

**Figure 1:**
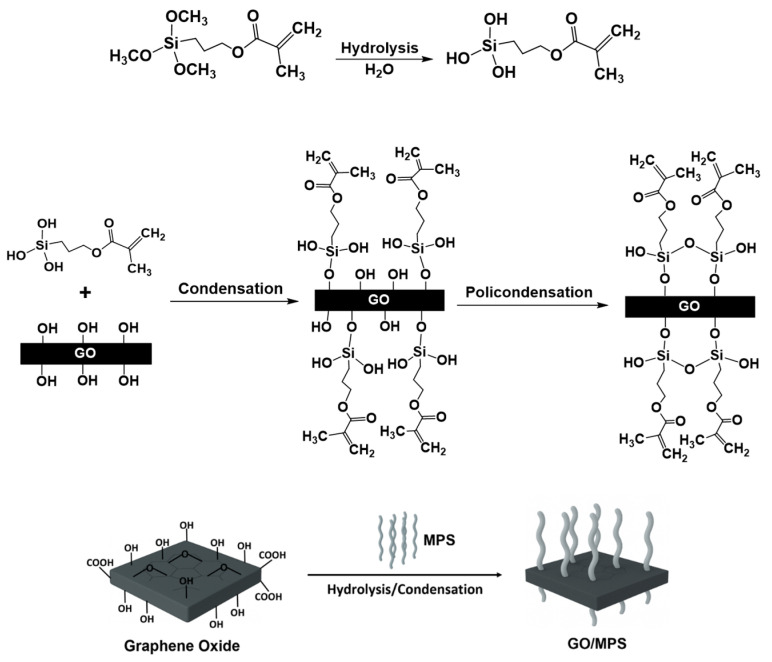
Schematic representation of the functionalisation of GO with silane groups via hydrolysis, condensation, and polycondensation reactions.

To prepare the hybrid material, graphene oxide (GO) was first functionalised with MPS to introduce vinyl groups capable of promoting covalent polymer grafting (Figure 1).

**Figure 2:**
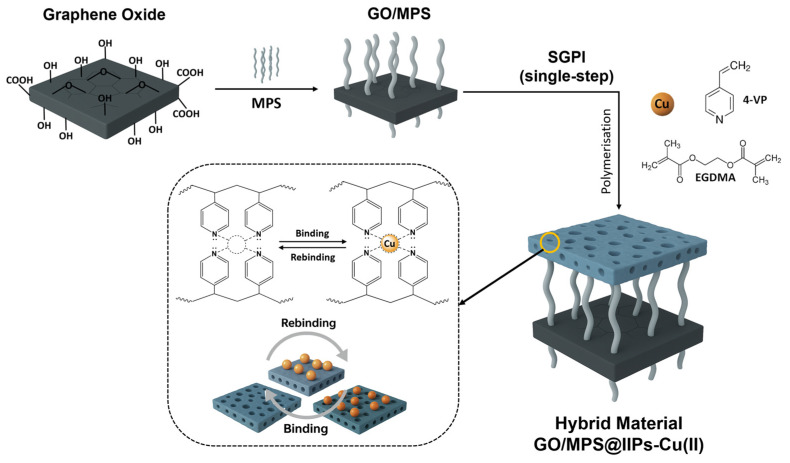
Schematic representation of the single-step synchronous grafting–imprinting–polymerisation (SGPI) route applied to MPS-functionalised GO for the preparation of the GO/MPS@IIPs-Cu(II) hybrid. The inset shows an enlarged view of the imprinted polymer layer, illustrating the binding and rebinding of Cu(II) ions within the recognition cavities.

The Cu(II)–4VP complex was then combined with the GO/MPS support, followed by the addition of EGDMA and AIBN. Under the synchronous grafting–imprinting–polymerisation (SGPI) strategy, complexation, polymer growth, and crosslinking proceeded simultaneously in a single step, promoting the formation of a surface-oriented imprinted polymer layer on the GO/MPS support (Figure 2).

Hybrid materials were synthesised using four different GO/MPS loadings (5, 10, 50, and 100 mg), while maintaining a constant 1:4:20 molar ratio. This approach enabled the influence of support on the imprinted architecture to be systematically evaluated without introducing additional compositional variables, allowing direct comparison between the bulk polymer and its hybrid counterparts. Among these formulations, the material containing 50 mg of GO/MPS exhibited the best overall adsorption performance in terms of adsorption capacity, kinetics, and selectivity. Therefore, this material is discussed in detail in the main text, whereas the remaining formulations are included in the Supplementary Information and only discussed when relevant.

Although the SGPI methodology integrates grafting and imprinting in a single step, consistent results were obtained across independently prepared batches. Similar FTIR spectra, comparable FESEM morphologies, and reproducible experimental results under identical conditions indicate reliable material formation. In this context, the degree of GO/MPS functionalisation and the dispersion of the support during polymerisation were identified as important factors influencing the final material structure, supporting the robustness of the SGPI approach.

### 3.2. Intermediates and Final Adsorbent Characterisation

The structural and morphological characterisation was performed on the ion-imprinted polymer IIPs-Cu(II) and its non-imprinted counterpart (NIP), the GO/MPS support, and the hybrid GO/MPS@IIPs-Cu(II) adsorbent and its corresponding NIP. FTIR, Raman spectroscopy, FESEM, and EDS analyses collectively provide evidence of template–monomer interactions, GO functionalisation, polymer grafting, and effective removal of the template ion.

#### 3.2.1. FTIR Analysis

**Figure 3:**
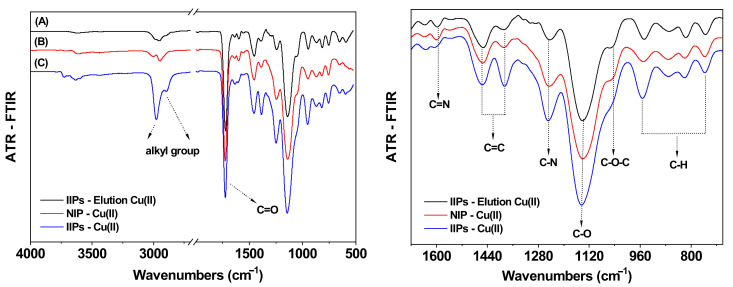
ATR–FTIR spectra of (A) IIPs-Cu(II) after template removal, (B) NIP, and (C) IIPs-Cu(II). (**Left**) Full spectral range (4000–500 cm^−1^); (**Right**) magnified region showing characteristic bands associated with C=O, C–N, and C–O groups.

The FTIR spectra of the IIPs-Cu(II) polymer (before and after template removal) exhibited the characteristic bands of the crosslinked polymer network (Figure 3), including C–H stretching vibrations (2950–2870 cm^−1^), the C=O stretching of EGDMA (1725 cm^−1^), and C–O vibrations associated with the ethylene glycol backbone (1145 and 1050 cm^−1^), confirming the successful formation of the polymer network [39,40].

The bands attributed to 4VP—C=N (~1600 cm^−1^) and C–N (1249 cm^−1^)—decreased in intensity after template elution, indicating their involvement in coordination interactions with Cu(II) during the imprinting process. The increased intensity of the band at 1456 cm^−1^ in the presence of the template ion further supports the formation of the Cu(II)–4VP complex [41,42]. In contrast, out-of-plane aromatic vibrations remained essentially unchanged after elution, indicating that the polymer framework was preserved following HNO_3_ treatment.

**Figure 4:**
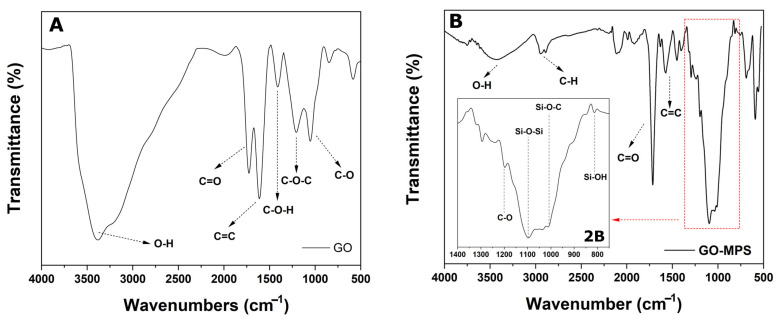
FTIR spectra of (**A**) graphene oxide (GO) and (**B**) MPS-functionalised graphene oxide (GO/MPS). Inset (**2B**) shows an enlarged section of the spectrum between 1400 and 800 cm^−1^, highlighting characteristic vibrational bands.

The FTIR spectrum of GO displayed the typical features of oxidised graphene materials, including O–H, C=O, aromatic C=C, and C–O–C vibrations [43]. After functionalisation with MPS, the appearance of Si–O–C and Si–O–Si bands (1099 and 1031 cm^−1^) [44,45,46], together with the attenuation of the O–H stretching band, confirms the successful covalent modification of the GO surface (Figure 4).

The FTIR spectrum of GO/MPS@IIPs-Cu(II) showed contributions from both the functionalised GO support and the imprinted polymer phase. Although partial band overlap is expected due to the coexistence of multiple functional groups, the overall spectral features are consistent with the successful formation of a hybrid structure in which the polymer is effectively grafted onto the GO/MPS surface. After template removal, the persistence of the main characteristic bands indicates that the structural integrity of the imprinted sites is largely preserved within the hybrid matrix.

#### 3.2.2. FESEM Analysis

**Figure 5:**
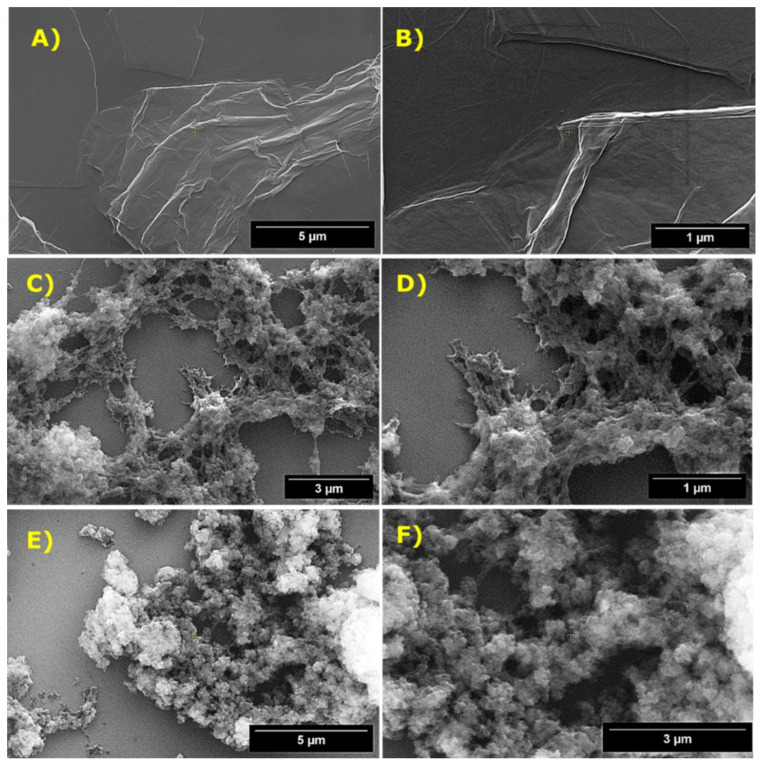
FESEM micrographs of (**A**,**B**) graphene oxide (GO), (**C**,**D**) bulk IIPs-Cu(II), and (**E**,**F**) hybrid GO/MPS@IIPs-Cu(II).

The FESEM micrographs of GO (Figure 5A,B) show its characteristic laminar morphology, with ripples and wrinkles commonly associated with partially exfoliated graphene-based materials. This morphology is consistent with oxidised graphene derivatives and reflects the presence of oxygen-containing functional groups that disrupt the sp^2^ lattice, as well as mechanical stresses introduced during oxidation and drying [25,47].

In the case of the bulk IIPs-Cu(II) polymer, the images (Figure 5C,D) reveal dispersed particles with a markedly rough and heterogeneous surface. The polymer exhibits a porous and irregular morphology, characteristic of highly crosslinked matrices produced via bulk free-radical polymerisation [48]. The presence of pores across different size ranges and the low packing density of polymer aggregates suggest an open structure that facilitates mass transfer towards internal adsorption sites [49].

In contrast, the hybrid GO/MPS@IIPs-Cu(II) material (Figure 5E,F) exhibits a more cohesive and compact morphology. The polymer phase appears as a continuous coating over the GO sheets, indicating that the functionalised graphene oxide directs the organisation of the polymer during growth. Polymer domains are more densely packed and structurally interconnected, suggesting that GO acts as a scaffold that promotes a more organised polymer assembly and stabilises the hybrid architecture [50]. The consistent formation of this compact structure across the material further supports effective integration of the GO/MPS support within the polymer network.

From a functional perspective, this organised morphology is consistent with enhanced structural stability and improved exposure of imprinted binding sites located at or near the surface. These features, together with the inherent surface roughness of the hybrid material, support the enhanced adsorption performance observed experimentally for the hybrid material and are in agreement with trends reported for similar surface-imprinted systems [51].

#### 3.2.3. Raman Analysis

In graphene-based materials, the intensity ratio between the D and G bands (ID/IG) provides insight into the degree of structural disorder in the sp^2^ carbon lattice. Values below unity are typically associated with partially preserved graphitic domains, whereas values approaching or exceeding unity indicate increased defect density and fragmentation of these domains [52,53]. The Raman spectrum of GO (Figure S1) exhibited the characteristic D (1351 cm^−1^) and G (1598 cm^−1^) bands, associated with structural defects and ordered sp^2^ regions, respectively. The resulting ID/IG ratio of 0.86 is consistent with partially oxidised graphene oxide, where defect-rich regions coexist with residual graphitic domains.

After functionalisation with MPS, the ID/IG ratio increased to 0.98, indicating an increase in defect density, consistent with covalent grafting onto the GO surface [54]. The positions of the D and G bands remained essentially unchanged, indicating that the basal graphitic framework is largely preserved following silanisation.

The 2D band, observed as weak and broadened in both materials, is characteristic of reduced crystalline order and reduced sp^2^ domain size in oxidised graphene. Its persistence after functionalisation indicates that small graphitic regions remain within the modified structure. 

#### 3.2.4. EDS Analysis

**Figure 6:**
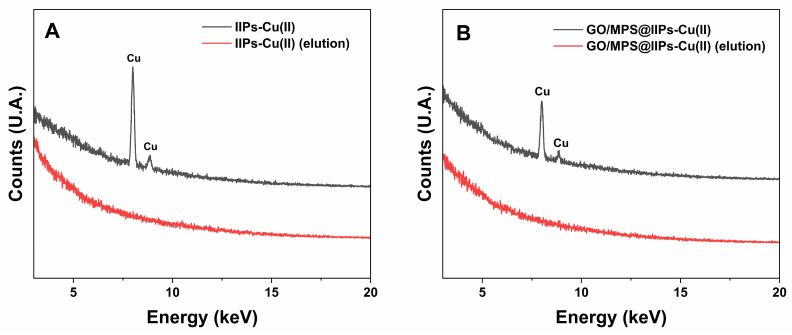
EDS spectra of (**A**) IIPs-Cu(II) and (**B**) GO/MPS@IIPs-Cu(II) before and after template removal.

EDS analysis was performed to verify the presence of Cu(II) in the imprinted polymers before and after template removal. In both the bulk IIPs-Cu(II) and the hybrid GO/MPS@IIPs-Cu(II) prior to elution, characteristic copper signals were clearly detected, confirming the successful incorporation of the template ion within the polymer matrix (Figure 6).

After elution, a marked decrease in the Cu signal was observed for both materials, with no significant residual copper detected, confirming the effective removal of the template ion. The elution behaviour of the hybrid material closely matched that of the bulk IIPs-Cu(II) polymer, demonstrating that the presence of the GO/MPS support does not hinder template extraction or the regeneration of the imprinted cavities.

These results are consistent with the FTIR and FESEM analyses, which indicate that both the chemical structure and morphological integrity of the materials are preserved after template removal. This structural stability is essential for maintaining the functionality of the imprinted binding sites and ensuring reliable adsorption performance during subsequent experiments.

### 3.3. Adsorption Studies

#### 3.3.1. Effect of pH

**Figure 7:**
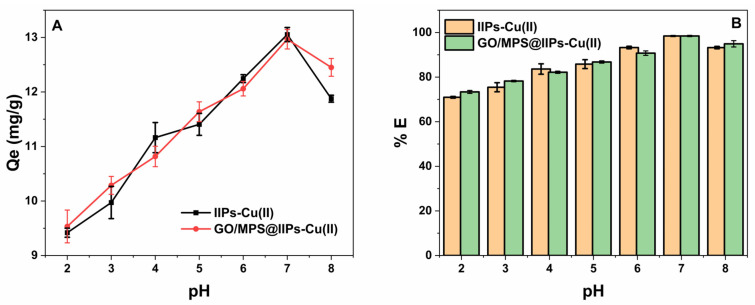
Effect of pH on the adsorption of Cu(II) onto IIPs-Cu(II) and GO/MPS@IIPs-Cu(II). (**A**) Adsorbed amount (Qe, mg g^−1^) as a function of pH. (**B**) Extraction efficiency (%E) at different pH values. Experimental conditions: 30 mg of adsorbent, initial Cu(II) concentration of 20 mg L^−1^, solution volume of 20 mL, shaking time of 1 h at 400 rpm, and room temperature. Data are presented as mean ± standard deviation (n = 3).

The effect of pH on the adsorption behaviour of IIPs-Cu(II) and GO/MPS@IIPs-Cu(II) was evaluated over the pH range 2–8. Figure 7 presents the variation in the adsorbed amount (Qe) and extraction efficiency (%E) as a function of pH, while the corresponding values are summarised in Table 1.

The adsorption response of both materials exhibited a strong dependence on pH, owing to its influence on the protonation state of functional groups and the aqueous speciation of Cu(II). Within the pH range of 2–7, a progressive increase in the Qe was observed for both materials (Figure 7A), reaching the highest values at pH 7. Similarly, the extraction efficiency (Figure 7B) increased from approximately 70–75% at pH 2 to ~98–99% at pH 7, indicating nearly complete removal of Cu(II).

At low pH values, the reduced Qe can be attributed to the protonation of functional groups, particularly pyridinic nitrogen sites, which diminishes their ability to coordinate Cu(II). This effect is compounded by competition between H^+^ ions and Cu(II) for the available binding sites. As the pH increased, progressive deprotonation enhanced the availability of electron-donor groups, thereby promoting stronger coordination interactions with Cu(II) ions [55].

Although both materials exhibited comparable adsorption responses across the studied pH range, the hybrid material achieved similar or slightly higher adsorbed amounts under specific experimental conditions. This behaviour may be associated with the presence of oxygen-containing groups derived from graphene oxide and the surface-localised distribution of imprinted sites generated through the SGPI strategy, which could facilitate the interaction of Cu(II) ions with the binding sites.

At pH 8, a slight decrease in the Qe was observed, more pronounced for the bulk polymer. This behaviour may be attributed to the formation of hydroxylated Cu(II) species, which reduce the fraction of free Cu(II) available for coordination. Nevertheless, the relatively moderate decrease, particularly for the hybrid material, suggests that adsorption is likely the dominant process under these conditions, consistent with the aqueous speciation of Cu(II) [56].

Throughout the experiments, the pH was verified after equilibration, and no visible turbidity or precipitation was observed, suggesting that adsorption remained the dominant removal process under the studied conditions. Based on these results, pH 7 was selected as the optimum condition for the subsequent kinetic and equilibrium studies.

#### 3.3.2. Adsorption Kinetics

**Figure 8:**
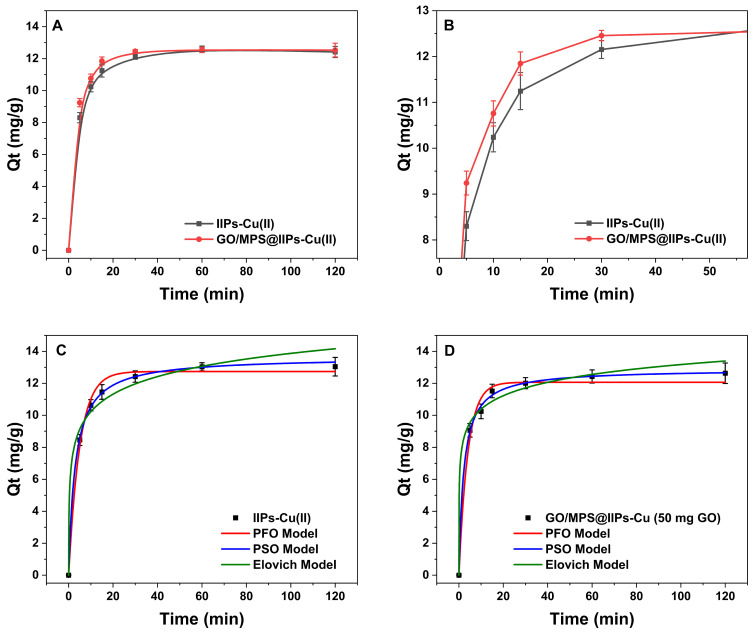
Adsorption kinetics of Cu(II) onto IIPs-Cu(II) and GO/MPS@IIPs-Cu(II). (**A**) Experimental kinetic profiles for both adsorbents; (**B**) magnified view of the initial kinetic region; (**C**) kinetic model fits for IIPs-Cu(II); and (**D**) kinetic model fits for GO/MPS@IIPs-Cu(II) (50 mg GO/MPS). Experimental conditions: 30 mg of adsorbent, initial Cu(II) concentration of 20 mg L^−1^, solution volume of 20 mL, pH 7, and room temperature. Data are presented as mean ± standard deviation (n = 3).

The adsorption kinetics of Cu(II) onto the synthesised adsorbents are presented in Figure 8. Figure 8A,B shows the experimental kinetic profiles for IIPs-Cu(II) and GO/MPS@IIPs-Cu(II), whereas Figure 8C,D presents the corresponding kinetic model fits obtained using the pseudo-first-order (PFO), pseudo-second-order (PSO), and Elovich models.

As shown in Figure 8A, both materials exhibited rapid initial uptake followed by a gradual approach to equilibrium. Equilibrium was considered reached when consecutive sampling times showed comparable Qe values within the experimental standard deviations. Notably, GO/MPS@IIPs-Cu(II) reached a near-equilibrium plateau after approximately 30 min, whereas IIPs-Cu(II) required around 60 min to reach a comparable level of stabilisation. This difference is more evident in the magnified view of the initial kinetic region (Figure 8B), where the hybrid material displayed a slightly faster kinetic response. Although these differences are relatively small and should be interpreted within the bounds of experimental variability, they suggest a modest improvement in the initial adsorption kinetics of GO/MPS@IIPs-Cu(II). This behaviour may be ascribed to the enhanced accessibility of surface-oriented imprinted sites generated through the SGPI strategy. Accordingly, the GO/MPS support is likely to facilitate the adsorption process at the early stages of contact by promoting more efficient interactions between Cu(II) ions and the imprinted binding sites [20,22].

Experimental kinetic data were fitted to pseudo-first-order (PFO), pseudo-second-order (PSO), and Elovich models, and the corresponding kinetic parameters are summarised in Table 2.

For both materials, the PSO model provided the best statistical fit, as indicated by the highest R^2^ values and the lowest χ^2^ values. The PSO rate constant (k_2_) obtained for GO/MPS@IIPs-Cu(II) (0.038 ± 0.004 g mg^−1^ min^−1^) was higher than that for IIPs-Cu(II) (0.027 ± 0.002 g mg^−1^ min^−1^), which is consistent with the kinetic behaviour observed for the hybrid material. Although the PFO model also yielded satisfactory fits (R^2^ > 0.999), its elevated χ^2^ values suggest a less accurate representation of the adsorption process. In contrast, the Elovich model exhibited poorer fitting performance, particularly for the hybrid material, as reflected by higher χ^2^ values and large uncertainties associated with parameter A.

Despite the excellent statistical agreement with the PSO model, it should be regarded as an empirical representation of the adsorption process rather than definitive evidence of a specific kinetic mechanism. Nevertheless, the close agreement with the PSO model is consistent with adsorption involving specific interactions between Cu(II) ions and the imprinted binding sites of the adsorbent [57].

#### 3.3.3. Adsorption Isotherms

**Figure 9:**
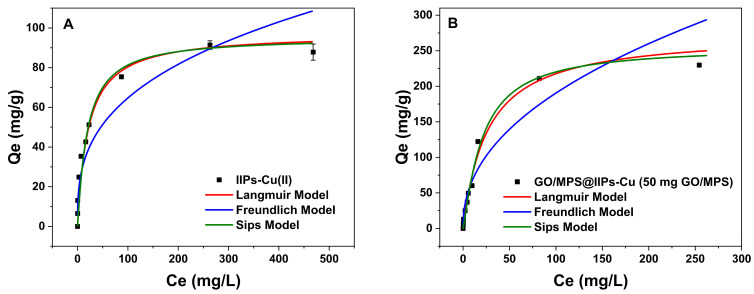
Equilibrium adsorption isotherms and nonlinear fitting using the Langmuir and Sips models and the Freundlich equation for Cu(II) adsorption onto IIPs-Cu(II) (**A**) and GO/MPS@IIPs-Cu(II) (**B**).

Isotherm analysis revealed a significant enhancement in adsorption performance upon incorporation of GO/MPS into the imprinted polymer matrix. The bulk IIPs-Cu(II) exhibited an experimental adsorbed amount of 92 mg g^−1^, whereas the GO/MPS@IIPs-Cu(II) hybrid prepared with 50 mg of GO/MPS reached 234 mg g^−1^ under the studied conditions (Figure 9), supporting the beneficial effect of the hybrid architecture on Cu(II) uptake. Furthermore, the Qmax values obtained from isotherm modelling exceeded those reported for conventional Cu(II)-imprinted polymers [58,59].

The equilibrium data were fitted using the Langmuir and Sips models and the Freundlich equation [60]. Among them, the Langmuir and Sips models provided the most accurate description for both materials, as evidenced by higher R^2^ values and lower χ^2^ values, indicating adsorption behaviour consistent with relatively well-defined binding sites with slight deviations from ideal homogeneity, in agreement with previously reported behaviour for ion-imprinted systems [61,62]. The corresponding nonlinear isotherm parameters and statistical fitting values are summarised in Table 3.

For bulk IIPs-Cu(II), both models showed comparable fitting quality (Langmuir: R^2^ = 0.9944, χ^2^ = 0.6; Sips: R^2^ = 0.9938, χ^2^ = 0.7), with predicted Qmax values (97 ± 2 and 95 ± 4 mg g^−1^, respectively) in close agreement with the experimental adsorbed amount, supporting a relatively well-defined distribution of imprinted sites. For the hybrid material, the Sips model provided a slightly improved fit (R^2^ = 0.9940; χ^2^ = 1.9) compared to the Langmuir model (R^2^ = 0.9931; χ^2^ = 2.2), suggesting the presence of moderate energetic heterogeneity associated with the hybrid structure. The Sips model predicted a Qmax of 256 ± 15 mg g^−1^, while the Langmuir model estimated a higher value (275 ± 13 mg g^−1^), both exceeding the experimental adsorbed amount. This behaviour suggests that, despite the increased density of adsorption sites, certain imprinted cavities may remain partially inaccessible under experimental conditions, most likely due to structural constraints within the hybrid network, as is commonly observed in heterogeneous imprinted systems [63,64]. The n_LF_ parameter remained close to unity (1.1 ± 0.1 for the bulk polymer and 1.2 ± 0.1 for the hybrid), suggesting relatively homogeneous adsorption behaviour with only limited heterogeneity introduced by the incorporation of GO. Increasing the GO/MPS content to 100 mg resulted in a high adsorption capacity (Qmax = 228 ± 17 mg g^−1^, Sips model), although slightly lower than that obtained for the 50 mg formulation. The corresponding n_LF_ value (0.95 ± 0.09) suggests a slight increase in heterogeneity, which may be associated with structural rearrangements at higher support loadings that reduce the effective accessibility of imprinted sites.

Formulations containing lower GO/MPS loadings (5 and 10 mg) exhibited reduced adsorption capacities compared with the 50 mg material (Table S1), indicating that an insufficient amount of support limits the effective exposure of binding sites. In contrast, excessive GO/MPS content may lead to partial aggregation or structural constraints within the polymer phase, thereby decreasing the overall adsorption efficiency [20,55].

Overall, these results demonstrate that the adsorption behaviour is strongly dependent on the GO/MPS content, with the 50 mg formulation providing an optimal balance between site accessibility, structural organisation, and adsorption capacity [65].

#### 3.3.4. Selectivity Studies

Selectivity was evaluated in binary Cu(II)/Co(II), Cu(II)/Fe(III), and Cu(II)/Ba(II) systems to assess the affinity of the adsorbents towards competing cations with different ionic radii and coordination preferences. Similar competitive adsorption behaviour has been reported for Cu(II)-imprinted systems [61,62].

The GO/MPS@IIPs-Cu(II) hybrid exhibited a significantly higher distribution coefficient for Cu(II) (Kd = 9210 mL g^−1^) than that observed for the bulk IIPs-Cu(II) (Kd = 3345 mL g^−1^), indicating an enhanced apparent affinity towards the target ion. In the Cu(II)/Co(II), Cu(II)/Fe(III), and Cu(II)/Ba(II) systems, the hybrid material showed markedly higher selectivity coefficients (k = 17.1, 22.6, and 83.2, respectively) compared with the bulk polymer (k = 6.5, 10.5, and 19.3), confirming improved recognition of Cu(II) over competing ions [59]. The corresponding distribution and selectivity coefficients for both materials are summarised in Table 4.

This selective discrimination is particularly pronounced for Ba(II), reflecting strong discrimination against an alkaline-earth cation with limited affinity for coordination with nitrogen-donor ligands. In contrast, the lower selectivity observed for Co(II) and Fe(III) is consistent with their ability to engage in coordination interactions, although with geometries and ligand-field preferences that differ from those of Cu(II).

From a chemical standpoint, this selective response can be rationalised by the complementarity between the preferred coordination environment of Cu(II) and the nature of the imprinted sites generated from 4VP. As a d^9^ metal centre, Cu(II) exhibits Jahn–Teller distortion, favouring well-defined equatorial coordination with nitrogen-donor ligands [38]. In this context, pyridinic imprinted cavities are likely to preserve the coordination environment established during the pre-polymerisation stage. In contrast, competing cations such as Co(II) and Fe(III) exhibit different coordination geometries and ligand-field stabilisation energies, resulting in lower affinity for these sites. Discrimination against Ba(II) is even more pronounced, as this cation interacts primarily through electrostatic interactions rather than directional coordination, in contrast to Cu(II), which forms partially covalent interactions with nitrogen-donor ligands. These results indicate that the imprinting process encodes both geometric and electronic complementarity within the binding sites.

The relative selectivity coefficients (k′), obtained from comparison with non-imprinted polymers (NIPs), were consistently higher than unity, confirming that the observed selectivity arises predominantly from the imprinted cavities rather than from non-specific interactions [55]. This effect is particularly evident for the hybrid material, where values are significantly enhanced, indicating that the incorporation of GO/MPS improves the expression of selective binding sites.

The incorporation of GO/MPS also contributes to improved accessibility of the imprinted cavities, as reflected in the progressive increase in Cu(II) distribution coefficients (Kd values) with increasing GO content. However, an optimal support loading was observed, as the material containing 50 mg of GO/MPS exhibited the highest selectivity, while a further increase to 100 mg did not lead to a proportional improvement. This behaviour suggests that excessive GO content may induce structural rearrangements within the hybrid network, partially reducing the effective exposure of recognition sites, as also reported for supported imprinted sorbents [66].

#### 3.3.5. Desorption and Reusability Studies

**Figure 10:**
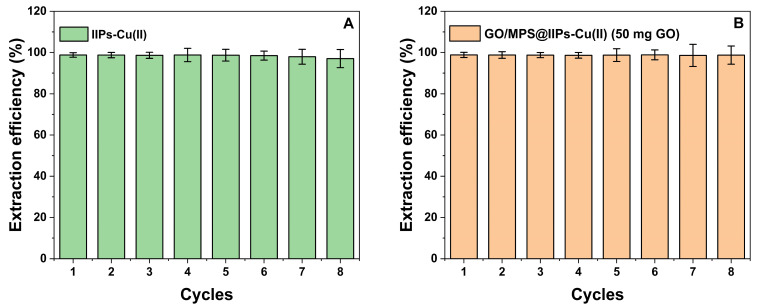
Reusability performance over eight consecutive adsorption–desorption cycles for (**A**) IIPs-Cu(II) and (**B**) GO/MPS@IIPs-Cu(II) (50 mg GO). Experimental conditions: 30 mg of adsorbent, initial Cu(II) concentration of 20 mg L^−1^, volume 20 mL, pH 7, and room temperature; desorption was carried out using 10% HNO_3_. Results are expressed as extraction efficiency (%). Data are presented as mean ± standard deviation (n = 4).

The operational stability of the adsorbents was evaluated over eight consecutive adsorption–desorption cycles using 10% HNO_3_ as the eluting agent. Both materials consistently maintained extraction efficiencies across all cycles (Figure 10), indicating reliable performance under repeated reuse conditions.

The GO/MPS-based material maintained nearly constant extraction efficiencies (≈98.6–98.9%), whereas IIPs-Cu(II) exhibited a minor decrease from ≈98.8% to ≈97.0% after repeated reuse. Although these differences were small and within the bounds of experimental variability, they suggest marginally greater stability for the hybrid material.

The slight decrease in extraction efficiency observed for the bulk polymer may be associated with gradual changes in the accessibility of imprinted sites during repeated adsorption–desorption cycles. In contrast, the surface-oriented architecture generated through the SGPI strategy is likely to promote a more organised distribution of recognition sites, thereby contributing to the preservation of adsorption efficiency upon reuse [13,16,22].

This interpretation is supported by FTIR and FESEM analyses, which confirmed the preservation of the chemical and morphological integrity of the materials. The reproducibility of the adsorption–desorption process was further supported by the relatively low RSD values (<6%), despite a slight increase at higher cycle numbers. Overall, these results highlight the potential of combining surface imprinting strategies with nanostructured supports for the development of robust and reusable adsorbent materials.

#### 3.3.6. Comparison of Adsorption Performance with Previously Reported GO-Based Cu(II)-Imprinted Systems

Adsorption capacities reported for Cu(II)-imprinted polymers vary considerably depending on the choice of functional monomer, crosslinking density, and polymer architecture. In unsupported IIPs, typical adsorption capacities range from 1.7 to 37 mg g^−1^, even when auxiliary ligands or mixed monomer systems (e.g., MAA/4VP) are employed. These relatively low values are generally attributed to the restricted accessibility of imprinted binding sites within highly crosslinked bulk polymer matrices (Table S2).

In this context, the bulk IIPs-Cu(II) synthesised in this work exhibited a Qmax value of 95 mg g^−1^, indicating that Cu–N coordination through the pyridinic nitrogen of 4VP enables the formation of effective binding sites without the need for auxiliary ligands, which may be partially lost during template removal. For supported systems, enhanced adsorption efficiency is typically associated with increased surface exposure of recognition sites. GO-, GO/MPS-, and magnetic GO-based IIPs generally exhibit Qmax values in the range of 109–192 mg g^−1^, often accompanied by faster sorption rates compared with bulk polymers.

The GO/MPS@IIPs-Cu(II) hybrid prepared via the SGPI strategy exhibited a Qmax of 256 mg g^−1^ at pH 7 and rapid adsorption kinetics, as confirmed by the isotherm and kinetic analyses. These results suggest that the material shows competitive performance compared with previously reported supported Cu(II)-imprinted polymers, offering a well-balanced combination of high adsorption capacity, improved adsorption kinetics, and satisfactory reusability, while placing it among the higher-performing GO-based systems reported to date.

A comparison with previously reported GO-based Cu(II)-imprinted systems is presented in Table 5.

Selectivity trends further reinforce this structural advantage. While unsupported IIPs often rely on auxiliary ligands that may undergo redistribution or partial loss after template removal, the bulk IIPs-Cu(II) exhibited competitive selectivity coefficients (k = 6.5 for Co(II), 10.5 for Fe(III), and 19.3 for Ba(II)). In contrast, the hybrid material showed substantially higher values (k = 17.1, 22.6, and 83.2, respectively), with relative selectivity coefficients (k′) ranging from approximately 6 to 24 depending on the competing ion (Table 4). These findings suggest that selectivity arises predominantly from well-defined imprinted cavities rather than from non-specific interactions.

A broader comparison with Cu(II)-imprinted systems reported in the literature, including both unsupported and GO-based materials, is provided in the Supplementary Information (Tables S3 and S4), confirming that the selectivity coefficients obtained in this work fall within the upper range typically reported for analogous systems.

Beyond numerical comparisons, structural aspects associated with different synthesis strategies must also be considered. Multistep approaches used to prepare supported IIPs may lead to heterogeneous coating thicknesses, partial encapsulation of binding sites, or irregular polymer growth. In contrast, the synchronous grafting–imprinting–polymerisation (SGPI) approach anchors the monomer–template complex directly onto the GO/MPS surface, directing polymer growth outward from the support and promoting a more accessible distribution of binding sites.

FESEM observations, together with the preservation of FTIR and Raman features, support the formation of a well-defined hybrid architecture. This controlled structure provides a rational basis for the enhanced adsorption performance, improved adsorption kinetics, selective recognition, and satisfactory reusability of GO/MPS@IIPs-Cu(II), positioning it as a competitive adsorbent relative to previously reported systems.

## 4. Conclusions

In this study, a hybrid adsorbent, GO/MPS@IIPs-Cu(II), was successfully synthesised through a single-step synchronous grafting–imprinting–polymerisation (SGPI) strategy, and its adsorption properties and selectivity towards Cu(II) were systematically evaluated. FTIR, Raman, FESEM, and EDS analyses confirmed the formation of a surface-oriented imprinted polymer layer anchored onto the GO/MPS support, together with the effective removal of the template ion.

The hybrid material exhibited a high Qmax of 256 mg g^−1^ and rapid adsorption kinetics, outperforming the bulk IIPs-Cu(II) in terms of adsorption performance and kinetic response. Isotherm analysis indicated that the Sips model adequately describes the adsorption process, suggesting a moderate degree of heterogeneity in the distribution of binding sites generated through the SGPI approach. Selectivity studies demonstrated preferential recognition of Cu(II) over competing ions (Co(II), Fe(III), and Ba(II)), as evidenced by elevated selectivity (k) and relative selectivity (k′) coefficients. Furthermore, the hybrid material maintained extraction efficiencies above 98% over eight consecutive adsorption–desorption cycles, indicating satisfactory operational stability and reusability.

These results indicate that the incorporation of GO/MPS, in combination with the SGPI strategy, enhances the accessibility and organisation of imprinted binding sites, thereby improving Cu(II) recognition under the studied conditions. While the findings are promising, further investigations are necessary to assess the performance of the adsorbent in real matrices and under more complex chemical environments.

Overall, the SGPI strategy establishes a versatile platform for the development of advanced adsorbent materials with potential extension towards other target metal ions. This versatility enables the projection of future applications in the selective recovery of valuable metals of limited availability, such as rare earth elements, as well as their possible integration into hydrometallurgical systems under dynamic operating conditions. In addition, the incorporation of functional ions (e.g., Ag^+^) could expand the scope of these materials towards specific applications, such as antimicrobial coatings for biofouling control.

## Figures and Tables

**Table 1:** Adsorbed amount (Qe) and extraction efficiency (%E) of Cu(II) at different pH values for IIPs-Cu(II) and GO/MPS@IIPs-Cu(II).

pH	IIPs-Cu(II)		GO/MPS@IIPs-Cu(II)	
	Qe (mg g^−1^)	%E	Qe (mg g^−1^)	%E
2	9.4 ± 0.1	71	9.5 ± 0.3	73
3	10.0 ± 0.3	76	10.3 ± 0.2	78
4	11.2 ± 0.3	84	10.8 ± 0.2	82
5	11.4 ± 0.2	86	11.6 ± 0.2	87
6	12.2 ± 0.1	93	12.1 ± 0.1	91
7	13.1 ± 0.1	98	13.0 ± 0.2	98
8	11.9 ± 0.1	93	12.5 ± 0.2	95

**Table 2:** Kinetic model parameters for Cu(II) adsorption onto IIPs-Cu(II) and GO/MPS@IIPs-Cu(II) (50 mg GO/MPS), obtained from pseudo-first-order (PFO), pseudo-second-order (PSO), and Elovich models.

Adsorbent	Model	Parameter 1	Parameter 2	χ^2^	R^2^	F-Value	Prob > F
IIPs-Cu(II)	PFO	k_1_ = 0.21 ± 0.02	Qe = 12.4 ± 0.2	1.48	0.9993	4233	<0.0001
	PSO	k_2_ = 0.027 ± 0.002	Qe = 13.27 ± 0.09	0.22	0.9999	28,287	<0.0001
	Elovich	A = 174 ± 217	B = 0.69 ± 0.12	4.14	0.9979	1512	<0.0001
GO/MPS@IIPs-Cu(II) (50 mg GO/MPS)	PFO	k_1_ = 0.26 ± 0.02	Qe = 12.5 ± 0.1	1.89	0.9995	6482	<0.0001
	PSO	k_2_ = 0.038 ± 0.004	Qe = 13.3 ± 0.1	0.91	0.9998	13,515	<0.0001
	Elovich	A = 1376 ± 2993	B = 0.9 ± 0.2	7.32	0.9981	1669	<0.0001

**Table 3:** Nonlinear isotherm parameters and statistical evaluation for Cu(II) adsorption onto IIPs-Cu(II) and GO/MPS@IIPs-Cu(II) using the Langmuir and Sips models and the Freundlich equation.

Adsorbent	Model	Parameter 1	Parameter 2	Χ^2^	R^2^	F-Value	Prob > F
IIPs-Cu(II)	Langmuir	Q_max_ = 97 ± 2	K_L_ = 0.047 ± 0.004	0.6	0.9944	1490	<0.0001
	Freundlich	K_F_ = 14 ± 3	n = 2.9 ± 0.4	7.6	0.9334	122	<0.0001
	Sips	Q_max_ = 95 ± 4	n_LF_ = 1.1 ± 0.1	0.7	0.9938	910	<0.0001
		K_LF_ = 0.04 ± 0.01					
GO/MPS@IIPs-Cu(II) (50 mg GO)	Langmuir	Q_max_ = 275 ± 13	K_L_ = 0.038 ± 0.004	2.2	0.9931	728	<0.0001
	Freundlich	K_F_ = 24 ± 5	n = 2.2 ± 0.3	24	0.9259	64	<0.0001
	Sips	Q_max_ = 256 ± 15	n_LF_ = 1.2 ± 0.1	1.9	0.9940	562	<0.0001
		K_LF_ = 0.029 ± 0.006					

**Table 4:** Distribution coefficient (Kd), selectivity coefficient (k), and relative selectivity coefficient (k′) for Cu(II) adsorption over competing ions using IIPs-Cu(II) and GO/MPS@IIPs-Cu(II).

Adsorbent	System	Kd (mL g^−1^)	k	Kd_NIP_ (mL g^−1^)	k_NIP_	k′
IIPs-Cu(II)	Cu(II)	3345	–	284	–	–
	Cu(II)/Co(II)	512	6.5	177	1.6	4.1
	Cu(II)/Fe(III)	320	10.5	155	1.8	5.7
	Cu(II)/Ba(II)	173	19.3	122	2.3	8.3
GO/MPS@IIPs-Cu(II) (50 mg GO)	Cu(II)	9210	–	459	–	–
	Cu(II)/Co(II)	540	17.1	166	2.8	6.2
	Cu(II)/Fe(III)	407	22.6	205	2.2	10.1
	Cu(II)/Ba(II)	111	83.2	134	3.4	24.3

**Table 5:** Comparison of GO-based Cu(II)-imprinted adsorbents reported in the literature, including functional monomers, modification strategies, adsorption capacity (Qmax), optimal pH, equilibrium time, and reusability (number of adsorption–desorption cycles).

Material	Monomer	Modification Strategy	Qmax (mg g^−1^)	pH	Time (min)	Reusability (Cycles)	Reference
GO/MPS-IIPs	Acrylamide	Silanisation grafting (MPS)	109.4	5.0	30	12	[30]
Fe_3_O_4_@GO	Acrylamide	Magnetic graphene oxide	129.9	7.0	–	5	[27]
GO/MPS-IIPs	Acrylamide	Silanisation grafting (MPS)	132.8	6.5	15	5	[29]
GO-IIPs	Acrylamide	Suspension polymerisation	192	5.0	90	5	[28]
GO/MPS@IIPs-Cu(II)	4VP	Silanisation grafting (MPS, SGPI approach)	256	7.0	30	8	This work

## Data Availability

Data are contained within the article. Further inquiries can be directed to the corresponding author.

## Supplementary Materials

The following supporting information can be downloaded at: https://www.mdpi.com/article/10.3390/polym18111362/s1, Figure S1: Raman spectra of GO and GO/MPS; Table S1: Adsorption isotherm parameters for Cu(II) uptake onto IIPs-Cu(II) and GO/MPS@IIPs-Cu(II) at different GO loadings, obtained from the Langmuir and Sips models and the Freundlich equation; Table S2: Comparison of unsupported Cu(II)-imprinted polymers; Table S3: Comparison of selectivity coefficients for Cu(II)-imprinted polymers; Table S4: Comparison of selectivity coefficients for GO-based Cu(II)-imprinted adsorbents.
