# Supplementary material for: Hybrid Graphene Oxide/Ion-Imprinted Polymer via Single-Step Grafting–Imprinting for High-Performance and Selective Cu(II) Adsorption
Source: Polymers (Basel). 2026 May 30;18(11):1362. doi: 10.3390/polym18111362 (PMC13259062; doi:10.3390/polym18111362)
Supplement: Supplementary file 1 [file polymers-18-01362-s001.zip › polymers-4301591-supplementary.pdf]

Supplementary Materials

This section includes additional characterization data, adsorption modelling results, and comparative analysis supporting the findings of this study.

Figure S1. Raman spectra of graphene oxide (GO) and MPS-functionalised graphene oxide (GO/MPS). The characteristic D and G bands are observed in both materials, along with a weak and broadened 2D band, indicating structural disorder and partial preservation of graphitic domains after functionalisation.

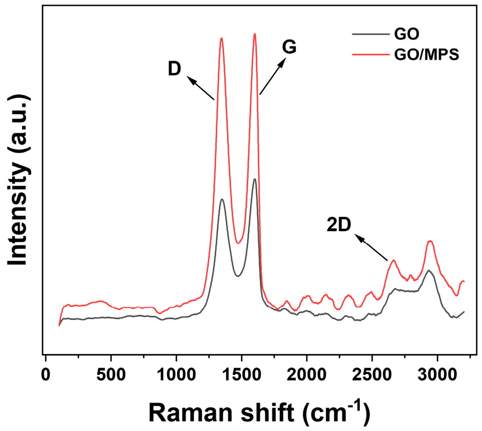

Table S1. Isotherm parameters for Cu(II) adsorption onto IIPs-Cu(II) and GO/MPS@IIPs-Cu(II) materials at different GO loadings, obtained from the Langmuir and Sips models and the Freundlich equation.

Adsorbent	Model	Parameter 1	Parameter 2	X^2	R^2	F-value	Prob > F
IIPs-Cu(II)	Langmuir	Q_{max} = 97 ± 2	K_L = 0.047 ± 0.004	0.6	0.9944	1490	<0.0001
	Freundlich	K_F = 14 ± 3	n = 2.9 ± 0.4	7.6	0.9334	122	<0.0001
	Sips	Q_{max} = 95 ± 4<br>K_{LF}= 0.04 ± 0.01	n_{LF} = 1.1 ± 0.1	0.7	0.9938	910	<0.0001
GO/MPS@IIPs-Cu(II) (5 mg GO)	Langmuir	Q_{max} = 79 ± 3	K_L = 0.10 ± 0.01	2.1	0.9947	846	<0.0001
	Freundlich	K_F = 6 ± 3	n = 2.2 ± 0.4	99	0.7455	14	0.0025
	Sips	Q_{max} = 77 ± 3<br>K_{LF}= 0.08 ± 0.03	n_{LF} = 1.2 ± 0.2	1.9	0.9952	633	<0.0001
GO/MPS@IIPs-Cu(II) (10 mg GO)	Langmuir	Q_{max} = 94 ± 3	K_L = 0.07 ± 0.01	2.1	0.9920	796	<0.0001
	Freundlich	K_F = 17 ± 3	n = 3.3 ± 0.4	10	0.9606	160	<0.0001
	Sips	Q_{max} = 105 ± 6<br>K_{LF}= 0.11 ± 0.02	n_{LF} = 0.71 ± 0.08	0.9	0.9961	1110	<0.0001
GO/MPS@IIPs-Cu(II) (50 mg GO)	Langmuir	Q_{max} = 275 ± 13	K_L = 0.038 ± 0.004	2.2	0.9931	728	<0.0001
	Freundlich	K_F = 24 ± 5	n = 2.2 ± 0.3	24	0.9259	64	<0.0001
	Sips	Q_{max} = 256 ± 15	n_{LF} = 1.2 ± 0.1	1.9	0.9940	562	<0.0001

		K_{LF}= 0.029 ± 0.006					
GO/MPS@IIPs-Cu(II) (100 mg GO)	Langmuir	Q_{max} = 221 ± 9	K_L = 0.048 ± 0.006	2.6	0.9903	515	<0.0001
	Freundlich	K_F = 16 ± 4	n = 2.1 ± 0.2	23	0.9125	54	<0.0001
	Sips	Q_{max} = 228 ± 17	n_{LF} = 0.95 ± 0.09	2.8	0.9894	315	<0.0001
		K_{LF}= 0.050 ± 0.008					

Table S2. Comparison of unsupported Cu(II)-imprinted polymers reported in the literature

Material	Monomer/Ligand	Qmax (mg g^{-1})	pH	Time (min)	Reusability (cycles)	Reference
IIPs-Cu(II)	4-(methacryloylamino)benzamide + 4-VP	1.7	6.0	60	10	[64]
IIPs-Cu(II)	4-VP + 4-(2-pyridylazo)resorcinol	4.4	7.0	20	≥100	[62]
IIPs-Cu(II)	4-VP + 2,2'-bipyridine	7.4	7.0	15	7	[58]
IIPs-Cu(II)	Methacrylic acid + 4-VP	14.9	~6.5	~100–120	–	[63]
IIPs-Cu(II)	Methacrylic acid + 4-VP	26.9	6.5	60	10	[59]
IIPs-Cu(II)	(2Z)-N,N′-bis(2-aminoethyl)but-2-enediamide (DBEDA)	29.8	4.0	30	20	[61]
IIPs-Cu(II)	Methacrylic acid + thiosemicarbazide	37.3	10	20	10	[65]
IIPs-Cu(II)	4-VP	95	7.0	60	8	This work

Table S3. Comparison of selectivity coefficients (k), selectivity coefficients for non-imprinted polymers (k_{NIP}), and relative selectivity coefficients (k′) reported for Cu(II)-imprinted polymers (IIPs) in the literature, including the material developed in this work.

Material	Selectivity coefficient (k)	Selectivity coefficient (k_{NIP})	Relative selectivity coefficient (k′)	Reference
IIPs-Cu(II)	Zn (36.8); Ni (79.6); Co (173.2)	Zn (1.4); Ni (2.5); Co (4.8)	Zn (26.3); Ni (31.8); Co (36.5)	[64]
IIPs-Cu(II)	Cd (163.3); Ni (163.3); Pb (245.0)	Cd (0.9); Ni (1.3); Pb (0.8)	Cd (181.4); Ni (125.6); Pb (306.3)	[62]
IIPs-Cu(II)	Ni (23.0); Co (4.3); Cd (10.5); Pb (7.2); Zn (4.3); Fe (10.5)	Ni (3.2); Co (3.9); Cd (2.5); Pb (2.5); Zn (2.0); Fe (2.0)	Ni (7.2); Co (1.1); Cd (4.2); Pb (2.9); Zn (2.2); Fe (5.4)	[58]

IIPs-Cu(II)	Ni (43.48); Zn (42.38)	Not reported	Not reported	[63]
IIPs-Cu(II)	Ni (16.5); Zn (13.8); Co (10.8); Fe (20.4)	Not reported	Not reported	[59]
IIPs-Cu(II)	Co (159.14); Ni (142.88); Zn (75.49); Cd (60.13)	Co (2.472); Ni (0.686); Zn (1.519); Cd (1.117)	Co (64.38); Ni (208.3); Zn (49.70); Cd (53.83)	[61]
IIPs-Cu(II)	Ni (14.1); Fe (42.3); Pb (32.4); Al (97.2); Cd (40.5); Zn (32.4); Ca (27.8); Mg (19.1); Co (16.8)	Ni (1.1); Fe (1.8); Pb (1.3); Al (3.4); Cd (1.4); Zn (2.1); Ca (1.5); Mg (1.3); Co (1.0)	Ni (12.8); Fe (23.5); Pb (24.9); Al (28.5); Cd (28.9); Zn (15.4); Ca (18.5); Mg (14.7); Co (16.8)	[65]
IIPs-Cu(II)	Co (6.5); Ba (19.3); Fe (10.5)	Co (1.6); Ba (2.3); Fe (1.8)	Co (4.1); Ba (8.3); Fe (5.7)	This work

Note: Selectivity coefficients were obtained under different experimental conditions depending on the source; therefore, comparisons should be considered qualitative.

Table S4. Comparison of selectivity coefficients (k), selectivity coefficients for non-imprinted polymers (k_{NIP}), and relative selectivity coefficients (k′) for GO-based Cu(II)-imprinted adsorbents reported in the literature, including the hybrid material developed in this work.

**Material**	**Selectivity coefficient (k)**	**Selectivity coefficient (k_{NIP})**	**Relative selectivity coefficient (k′)**	**Reference**
GO-IIPs-Cu(II)	Ni (39); Zn (27); Cd (60); Co (53)	Not reported	Not reported	[28]
GO/MPS-IIPs- Cu(II)	Zn (36.33); Co (77.3); Cd (8.5); Ni (8.44)	Zn (8.28); Co (4.37); Cd (5.7); Ni (5.82)	Zn (4.39); Co (17.69); Cd (1.50); Ni (1.45)	[29]
SiO_2–MPS@IIP–Cu(II)	Zn (160.7); Co (185.4); Ni (126.8); Fe (141.8); Mn (114.8); Cd (24.59); Hg (9.87); Al (8.64); Pb (9.49)	Zn (5.85); Co (6.23); Ni (6.04); Fe (5.56); Mn (4.50); Cd (2.80); Hg (1.19); Al (1.51); Pb (3.95)	Zn (27.46); Co (29.75); Ni (21.00); Fe (25.50); Mn (25.50); Cd (2.80); Hg (1.19); Al (1.51); Pb (2.40)	[66]
GO/MPS-IIPs-Cu(II)	Na (135.23); K (16.33); Zn (23.06); Ni (36.13); Co (45.93); Mn (58.81); Cd (18.13); Ca (66.78); Cr (17.85)	Na (12.22); K (1.84); Zn (3.16); Ni (3.88); Co (4.22); Mn (5.71); Cd (2.12); Ca (2.84); Cr (2.23)	Na (11.06); K (8.83); Zn (7.28); Ni (9.30); Co (10.88); Mn (10.30); Cd (8.53); Ca (23.43); Cr (7.98)	[30]
GO/MPS-IIPs-Cu(II)	Co (17.1); Ba (83.2); Fe (22.6)	Co (2.8); Ba (3.4); Fe (2.2)	Co (6.2); Ba (24.3); Fe (10.1)	This work

Note: Selectivity coefficients were obtained under different experimental conditions depending on the source; therefore, comparisons should be considered qualitative.
